# Evaluation of Cleaning Methods for Lithium Disilicate Ceramic Post Try-In Paste Application: An SEM Analysis

**DOI:** 10.3390/dj12090281

**Published:** 2024-08-31

**Authors:** Gildo Coelho Santos Junior, Maria Jacinta Moraes Coelho Santos

**Affiliations:** Schulich School of Medicine & Dentistry, Western University (UWO), London, ON N6G 4L1, Canada; msantos9@uwo.ca

**Keywords:** try-in paste, ceramic, veneer, cleaning methods

## Abstract

This in vitro study assessed the efficacy of three cleaning methods on lithium disilicate ceramic after the application of different try-in pastes through SEM analysis. Ten rectangular specimens of IPS e.max CAD were prepared using a diamond disc, crystallized, etched with 5% hydrofluoric acid, and subjected to three try-in pastes—Calibra ©, Variolink (V), RelyX Veneer^®^—and three cleaning techniques—air–water spray (RD), ultrasonic bath in distilled water for five minutes (ULT/W), and ultrasonic bath in distilled alcohol for five minutes (ULT/A). A control specimen was also included. After one-minute paste application and subsequent cleaning method application, SEM evaluation was conducted. The results indicate that RD was as effective as CTRL in removing remnants from R-RD, V-ULT/W and V-ULT/A samples, but ineffective for all Calibra paste-contaminated specimens. In conclusion, the optimal removal of try-in paste residues from lithium disilicate restorations is paste-dependent; however, ultrasonic baths with distilled water or alcohol proved effective for most pastes tested.

## 1. Introduction

The development of dental materials and techniques has markedly propelled the field of prosthodontics into the age of aesthetic dentistry, marked by the adoption of lithium disilicate (LD) ceramics. Celebrated for its superior mechanical properties, aesthetic qualities, and biocompatibility, LD ceramic has become the preferred choice for fabricating veneer restorations. Its unique composition, which includes quartz, lithium dioxide, phosphor oxide, alumina, potassium oxide, and additional elements, delivers both translucency and color stability that closely resemble natural teeth [1]. This makes LD ceramics ideal for producing veneers that are not only durable and visually appealing, but also amenable to conservative dental approaches and adhesive cementation techniques.

Advancements in cementation materials and bonding techniques have significantly extended the applications of ceramics, making ceramic veneers a proven method and a viable alternative to more invasive conventional restorative treatments [2]. The widely acknowledged acid etch-rinse system is considered the “gold standard” for enamel bonding [3,4,5]. For the surface treatment of porcelain veneers, hydrofluoric acid etching and silane coupling agents are the most effective methods [6,7,8,9]. This method involves critical steps such as hydrofluoric acid etching to achieve micro-mechanical adhesion and the application of silane to promote a chemical adhesion between the ceramic and resin cement [7,10,11]. Despite these advances, challenges persist, particularly in post-etching cleaning methods [12,13,14,15].

Recently, resin cement systems have been developed to create a strong and reliable bond between ceramic veneers and tooth structures. These systems are available in a wide range of colors, offering diverse hues, chroma, and values, which are essential for achieving an aesthetically pleasing result. In addition to their color-matching capabilities, these materials exhibit high fracture resistance and long-term durability, contributing to their satisfactory clinical performance [16,17]. Specifically designed for luting ceramic veneers, these resin cements are typically light-cured, which endows them with extended working time and enhanced color stability compared to chemically cured or dual-cured alternatives [18,19,20].

During the color evaluation process, the ceramic veneer is positioned over the tooth substrate before cementation, often using water, glycerin gel, or try-in pastes to simulate the final appearance [7]. The use of try-in pastes is particularly critical in achieving the desired aesthetic outcome; however, these pastes can compromise the surface integrity of the ceramic [21]. Composed of water-soluble glycerin, mineral additives, and colorants, try-in pastes must closely match the shade of the corresponding resin cement to allow both clinicians and patients to accurately assess the final color result after cementation and curing [22].

The ability to adjust and visualize color in ceramic veneers is largely attributed to the exceptional optical properties of monolithic silicate ceramics, particularly lithium disilicate. These materials are renowned for their superior translucency, which closely mimics the natural appearance of tooth enamel, making them ideal for aesthetic dental restorations. Lithium disilicate, in particular, stands out due to its ability to balance translucency with mechanical strength, allowing for precise color adjustments and enhanced visualization during the try-in phase. The material’s unique crystalline structure enables light transmission in a way that facilitates accurate color matching, ensuring that the final restoration blends seamlessly with the surrounding dentition. This optical versatility is one of the key reasons why lithium disilicate is widely regarded as a gold standard in aesthetic restorative dentistry [23].

While try-in pastes generally correspond to their resin cement counterparts across most color ranges, the thickness of the ceramic can significantly affect color perception [10]. Moreover, residues from these pastes can interfere with the adhesive properties of the ceramic, potentially compromising the bond strength. Therefore, a meticulous post-etching cleaning regimen is crucial to ensure optimal adhesion and the long-term success of the restoration [12,22,24,25]. This cleaning process is essential to remove any contaminants that might hinder the bonding process, thereby ensuring the durability and reliability of the ceramic veneer restoration.

Addressing cleaning concerns, various methods such as ultrasonic baths, air/water sprays, and acid etching have been explored [23,26,27,28]. However, their effectiveness varies, and there is ongoing debate regarding their potential impact on the ceramic surface, which could either improve or compromise the adhesive bond [25]. It has been observed that during intraoral try-in, exposure to contaminants such as saliva or blood can degrade the bond strength of LD ceramics [23,26].

The integrity of the bond between the ceramic veneer and tooth substrate is paramount, and it can be significantly influenced by the ceramic’s surface condition following the try-in step. This emphasizes the need for thorough cleaning protocols. Additionally, the application of acidic cleaning agents requires careful consideration due to the potential for surface alterations from acid etching, which may produce precipitated silica and fluorine salts that remain attached to the ceramic surface, compromising the bonding process [29].

Contamination during the try-in procedure, whether from saliva, blood, or residue from fitting indicators like silicone or try-in pastes, poses a significant challenge to achieving a strong bond with resin luting cements [30]. 

Previous studies have investigated the effects of some cleaning agents, including air/water spray, ultrasonic baths, and etching with acids, on the bond strength of resin cements to ceramic [23,24,25,27,28]; however, a definitive and universally accepted cleaning protocol for these materials remains elusive [29,31]. The manuscript aims to elucidate the impacts of different cleaning methods on the removal of try-in pastes from LD ceramics through detailed scanning electron microscopy (SEM) analysis, enhancing the understanding of surface interactions and their importance in adhesive dentistry. This research contributes to the body of knowledge, offering valuable clinical insights to optimize the use of LD ceramics in veneer restorations.

## 2. Materials and Methods

The study utilized ten rectangular specimens of lithium disilicate (IPS e.max CAD, Ivoclar Vivadent, Schaan, Liechtenstein), prepared using a precision cutting machine (Accutom-50b) with water-cooling. The blocks were ground flat using 600-grit silicon carbide paper (3M™ Wetordry™ Sandpaper, 32036, A-Dec, 2601 Crestview Drive, Newberg, OR, USA) under water-cooling conditions. Subsequently, the specimens underwent ultrasonic cleaning (Renfert Easyclean, Renfert, Hilzingen, Germany) for 5 min in distilled water to remove any residue. After drying with oil-free compressed air, they were sintered in the corresponding porcelain furnace (Programat P700, Ivoclar Vivadent, Schaan, Liechtenstein) following the manufacturer’s instructions. Following crystallization, the ceramic specimens were ultrasonically cleaned again for 5 min in distilled water. The specimens were etched with 5% HF gel (CEREC^®^ Ceramics Etch, VITA Zahnfabrik, Bad Säckingen, Germany) for 60 s, rinsed with water spray for 20 s, air-dried, and randomly subjected to contamination for one minute with one of three different try-in pastes, before being assigned to groups as per Table 1.

The specimens were organized into groups based on the type of try-in paste and cleaning method used, including a control group without contamination, as follows: CTRL; C-RD, C-ULT/W, C-ULT/A; V-RD, V-ULT/W, V-UL/A; R-RD, R-ULT/W, and R-ULT/A.

Following the cleaning process, the specimens were analyzed using scanning electron microscopy (SEM) to evaluate the surface morphology and microstructural characteristics, assessing the effectiveness of each cleaning method in removing try-in paste residues. 

In preparation for the SEM analysis, the samples were mounted on aluminum stubs using carbon tape to ensure good electrical conductivity and stability, and coated with a thin layer of conductive material (platinum), a crucial step to prevent the charging of the specimen surface, improve the electron signal, and enhance the quality of the images obtained during SEM examination. The coating was applied using a sputter coater, ensuring a uniform and conductive surface for detailed and accurate imaging of the specimen’s features, including any residual try-in paste.

The SEM analysis was conducted using a Hitachi SU8230 Regulus Ultra High-Resolution Field Emission, operating at an accelerating voltage of 10 kV. Specimens were examined under the SEM at a 5K magnification to identify and assess the presence of residual try-in paste. This procedure allowed for high-resolution imaging of the ceramic surfaces, providing detailed visual evidence of the effectiveness of each cleaning method in removing the try-in paste residues. The high magnification enabled the observation of even the smallest remnants of the paste, ensuring a thorough evaluation of the cleaning methods. By utilizing SEM, precise images were captured that highlighted the surface interactions and the degree of cleanliness achieved by each method. This detailed visual assessment is crucial for understanding how different cleaning protocols impact the surface characteristics of lithium disilicate ceramics, which in turn affect the bonding quality and long-term success of ceramic restorations.

For the image analysis, the presence of residual try-in paste on the ceramic surfaces was evaluated using a qualitative scoring system. This scoring was performed based on the amount of residual paste observed within the image frame at a magnification of 5000x. The scores were defined as follows:

None—No visible residual paste in the SEM image;
Low—Residual paste occupies less than 25% of the image frame;
Medium—Residual paste occupies 25% to 50% of the image frame;
High—Residual paste occupies 50% to 75% of the image frame;
Very High—Residual paste occupies more than 75% of the image frame.

Each sample was assigned a score based on these criteria to provide a standardized assessment of the effectiveness of the cleaning methods. This scoring system allows for a more consistent and quantifiable comparison between the different cleaning methods and try-in pastes used in this study.

## 3. Results

Table 2 presents the amount of residual paste and surface coverage percentage after different cleaning methods were applied to three types of try-in pastes: Calibra, Variolink, and Rely-X. The cleaning methods evaluated include Rinse and Dry, Rinse and Dry followed by an Ultrasonic Bath in Distilled Water, and Rinse and Dry followed by an Ultrasonic Bath in Alcohol. A control sample with no contamination was also included for comparison. The results demonstrate varying degrees of residual paste removal efficiency depending on the paste type and cleaning method used.

**Figure 1:**
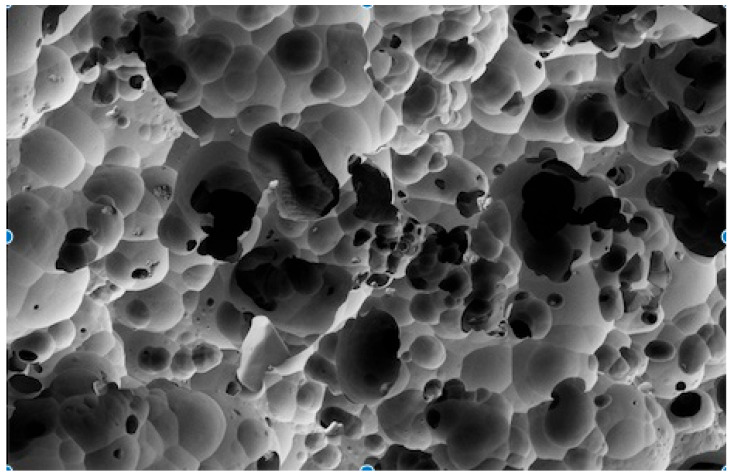
The control sample (No Contamination) showed a completely clean surface with no residual paste (Score: None).

**Figure 2:**
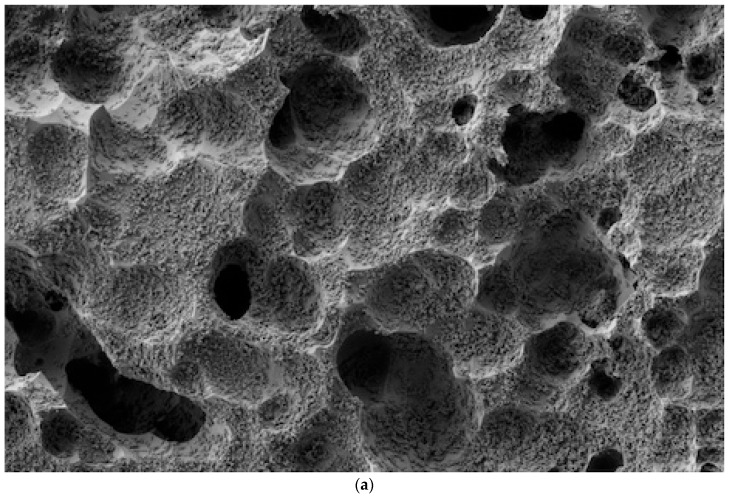

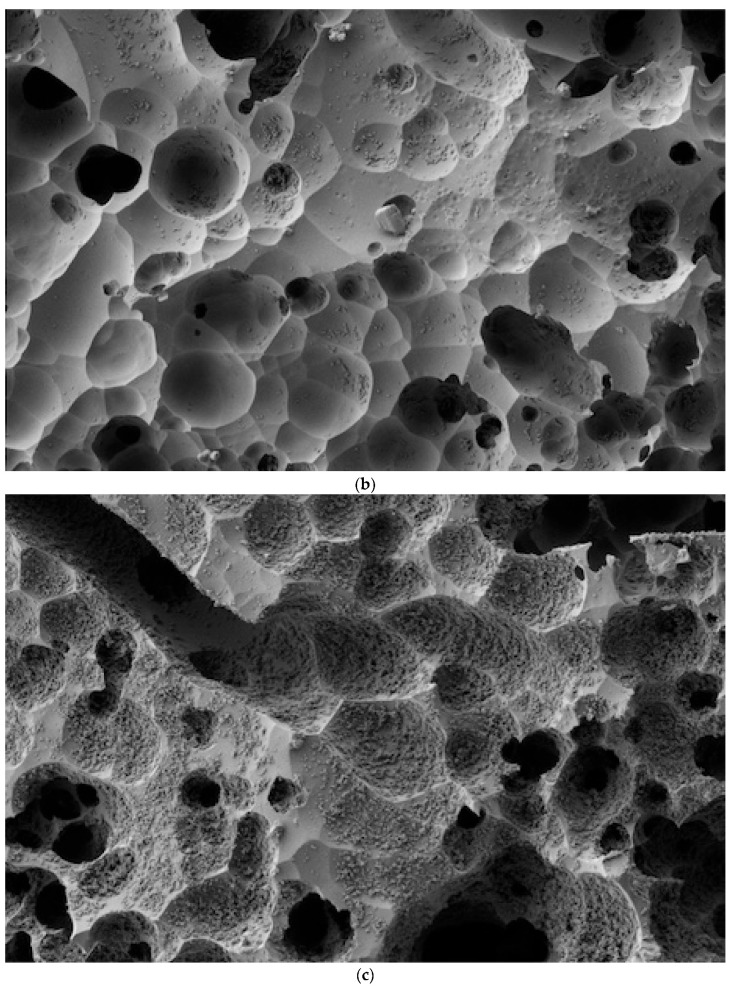
(**a**) The Calibra Try-in Paste cleaned with the Rinse and Dry method showed a very high amount of residual paste, occupying more than 75% of the image frame. (**b**) The Calibra Try-in Paste cleaned with Rinse and Dry followed by an Ultrasonic Bath in Distilled Water left a low amount of residual paste, covering less than 25% of the image frame. (**c**) The Calibra Try-in Paste cleaned with Rinse and Dry followed by an Ultrasonic Bath in Alcohol exhibited a very high amount of residual paste, similar to Rinse and Dry, occupying more than 75% of the image frame.

**Figure 3:**
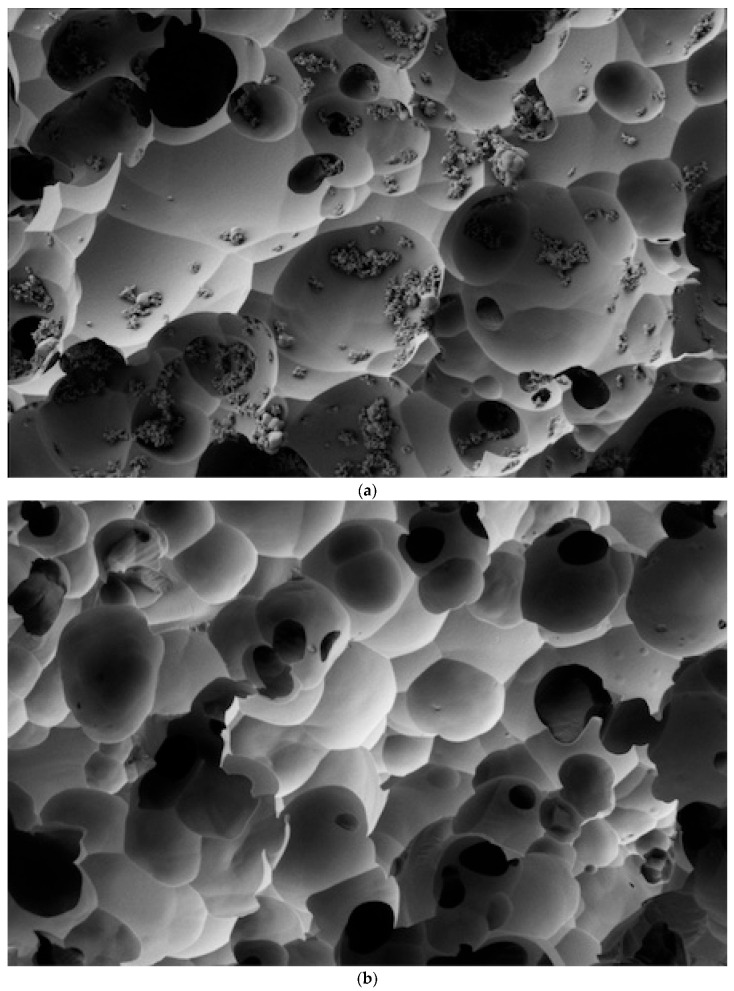

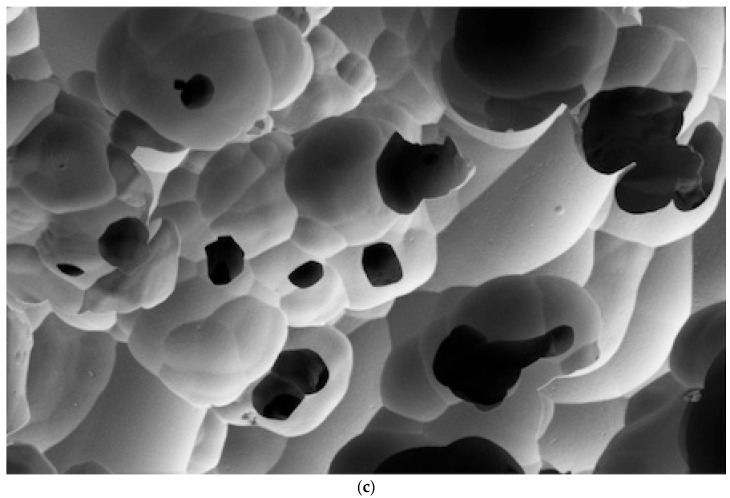
(**a**) The Variolink Try-in Paste cleaned with the Rinse and Dry method left a low amount of residual paste, covering less than 25% of the image frame. (**b**) The Variolink Try-in Paste cleaned with Rinse and Dry followed by an Ultrasonic Bath in Distilled Water showed a completely clean surface with no residual paste (Score: None). (**c**) The Variolink Try-in Paste cleaned with Rinse and Dry followed by an Ultrasonic Bath in Alcohol also showed a completely clean surface with no residual paste (Score: None).

**Figure 4:**
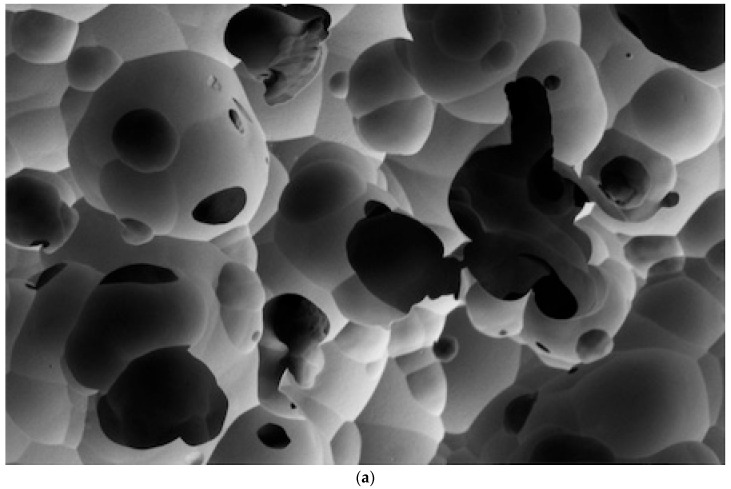

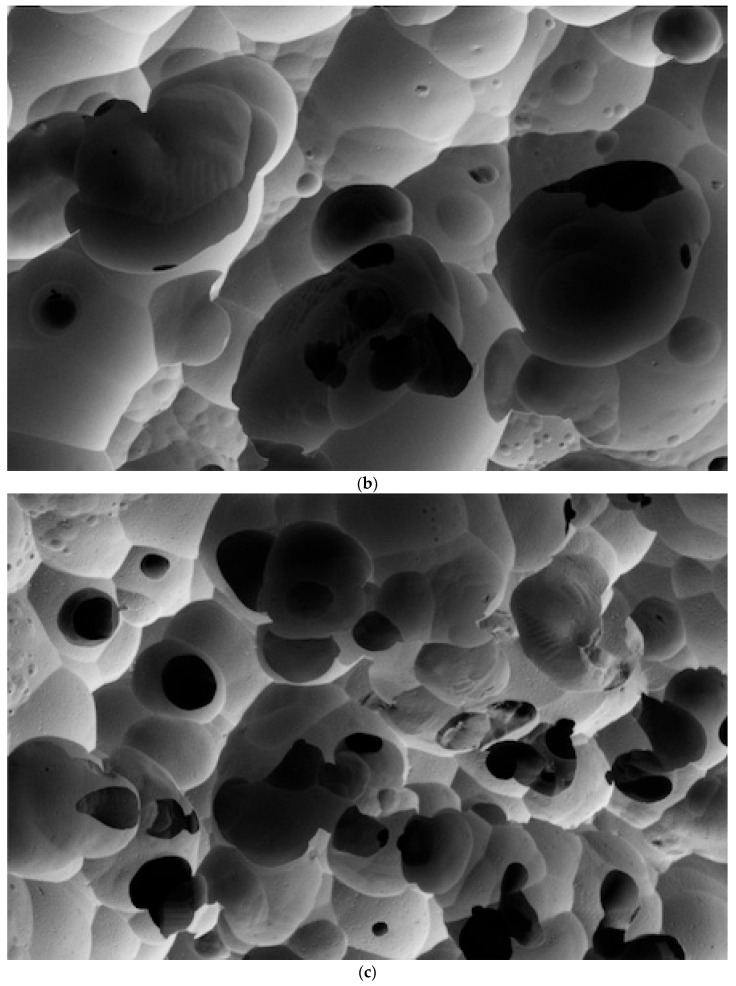
(**a**) The RelyX Veneer Try-in Paste cleaned with the Rinse and Dry method di-played a completely clean surface with no residual paste (Score: None). (**b**) The RelyX Veneer Try-in Paste cleaned with Rinse and Dry followed by an Ultrasonic Bath in Distilled Water showed a completely clean surface with no residual paste (Score: None). (**c**) The RelyX Veneer Try-in Paste cleaned with Rinse and Dry followed by an Ultrasonic Bath in Alcohol also showed a completely clean surface with no residual paste (Score: None).

The SEM analysis revealed clear differences in the effectiveness of various cleaning methods on lithium disilicate ceramic surfaces after contamination with different try-in pastes, as per Figure 1, Figure 2, Figure 3 and Figure 4. 

## 4. Discussion

In the realm of removing try-in paste and ensuring optimal adhesion between lithium disilicate ceramics and resin cements, the research landscape is diverse and evolving. Various cleaning methods have been proposed [23,24,27,28], albeit with varying efficacy, prompting questions about the potential for altered surface characteristics that could either enhance or compromise the adhesive bond [25].

Because hydrofluoric acid, which is commonly used to ensure macromechanical retention on the internal surfaces of glass ceramics, is highly corrosive, many clinicians delegate this step to the laboratory [29,32,33]. The present study aimed to investigate the cleaning methods’ influence on removing try-in pastes from LD ceramics, using scanning electron microscopy (SEM) analysis to provide a detailed examination of surface interactions and their possible implications for adhesive dentistry. The unavoidable contamination of LD during try-in, which could potentially weaken restoration bond strength, was also a common theme observed across studies and corroborated by our research findings [12,22,24,25].

As illustrated in Figure 1, Figure 2, Figure 3 and Figure 4, the control sample, which was not exposed to any contamination, presented a pristine surface with no residual paste, serving as the benchmark for evaluating the other samples.

When examining the effectiveness of the cleaning methods, the Rinse and Dry (RD) method demonstrated varying degrees of success depending on the type of try-in paste used. For the RelyX Veneer Try-in Paste, the RD method effectively removed the residues, resulting in a clean surface comparable to the control (Figure 4a). The application of ultrasonic baths, whether in distilled water or alcohol, also presented a cleaning efficacy, removing residual paste to undetectable levels (Figure 4b,c).

In contrast, the Variolink Try-in Paste showed moderate resistance to the RD method, with a medium amount of residual paste covering 25% to 50% of the surface (Figure 3a). However, when the RD method was followed by an ultrasonic bath, the amount of residual paste was significantly reduced to low levels, occupying less than 25% of the image frame (Figure 3b,c).

The most challenging paste to clean was the Calibra Try-in Paste, which exhibited a very high level of residual paste after cleaning with the RD method, covering more than 75% of the image frame (Figure 2a). Even with the addition of ultrasonic baths in distilled water or alcohol, the residual paste remained high, occupying 50% to 75% of the surface (Figure 2b,c). These findings indicate that the Calibra Try-in Paste requires more aggressive or alternative cleaning methods to achieve satisfactory results.

Overall, the results underscore the significant variability in cleaning efficacy based on the type of try-in paste and the cleaning method employed. While ultrasonic baths generally improved cleaning outcomes, their effectiveness varied significantly depending on the paste used. The RelyX Veneer Try-in Paste responded well to all cleaning methods, particularly when followed by ultrasonic baths, whereas the Calibra Try-in Paste presented substantial challenges. These observations highlight the importance of selecting appropriate cleaning protocols tailored to the specific materials used to ensure optimal surface preparation and adhesive bonding. The detailed findings and comparisons are clearly presented in the respective figures for a comprehensive review of the study’s outcomes.

Significant findings from the literature reveal a dearth of information on effective post-etching cleaning methods for LD ceramics, with one study demonstrating that a simple 30 s air/water spray could remove most residues effectively without damaging the material [12]. This aligns with our findings, which also underscore the practicality of such a method in clinical settings. Another study reported that groups treated with Ivoclean cleaning paste exhibited higher bond strengths and fewer failures compared to groups cleaned with air–water spray and 37% phosphoric acid. However, after aging, all cleaning methods performed similarly [22].

Moreover, an in-depth analysis from another study assessed the effectiveness of three distinct cleaning protocols, including 37% phosphoric acid, Ivoclean (sodium hydroxide, Restorative Cleaning Agent), and hydrofluoric acid, on the shear bond strength of LD restorations post-saliva and –silicone-disclosing medium (one alternative to try-in paste) [22]. The SEM results compared favorably with those of the present study, which similarly indicate that cleaning methods significantly influence the amounts of residual elements in each group using SEM analysis.

The present study validated the importance of SEM analysis in effectively evaluating the removal of try-in paste residues, contributing to refining cleaning protocols and underscoring the importance of meticulous surface preparation to ensure durable, reliable outcomes for ceramic restorations in dentistry. A limitation of the present study was not evaluating dedicated cleaning agents available on the market, such as Ivoclean (Ivoclar Vivadent), Monobond Etch & Prime (Ivoclar Vivadent), and other similar products. However, the goal of this study was to verify the effectiveness of simple and accessible methods for the complete removal of try-in paste on LD veneer ceramic. Regarding the use of SEM analyses, the primary objective of this study was to qualitatively assess the efficiency of different cleaning methods for lithium disilicate ceramics. The SEM analysis was chosen specifically for its ability to provide detailed visual insights into the surface interactions and to highlight the effectiveness of each cleaning method. The literature remains ambiguous regarding the quantitative assessments performed through mechanical tests, and the authors’ intention was to shine a light on the qualitative aspects, especially considering that the choice of cleaning method can significantly impact the bonding quality and the long-term success of ceramic restorations. We understand the importance of quantitative data and comprehensive statistical analysis in supporting the findings of scientific research. However, in this particular study, the qualitative insights provided by SEM analysis were crucial for understanding the surface characteristics post-cleaning, which directly influence the adhesion process. This approach allows the authors to offer clinicians practical, visually supported recommendations for optimizing cleaning protocols.

Future research should continue to explore the mechanistic aspects of paste–material interactions and refine cleaning protocols to guarantee predictable outcomes in adhesive dentistry, reinforcing the significance of thorough surface treatment methods.

## 5. Conclusions

Within the limitations of this in vitro study, it can be concluded that the effectiveness of try-in paste removal from lithium disilicate ceramics depends on the specific pastes used. The key findings include:

- Ultrasonic baths in distilled water or alcohol were generally effective, but outcomes varied with different try-in pastes tested;
- The Rinse and Dry method provided superior cleanliness for RelyX Veneer try-in paste compared to other try-in pastes tested;
- Ultrasonic baths in distilled water were effective for the removal of most try-in pastes, except for Calibra try-in paste, indicating that while this method is highly effective, it is not universally optimal.

## Figures and Tables

**Table 1:** SEM evaluation groups based on try-in paste and cleaning methods.

Group	Try-in Paste	Manufacturer	Cleaning Method
1	No Contamination (CTRL)		No Cleaning (Control)
2	Calibra Try-in Paste (**C**)	DENTSPLY Sirona, York, PA, USA	Air–Water Spray or Rinse and Dry (RD)
3	Calibra Try-in Paste (**C**)		Rinse and Dry + Ultrasonic Bath in Distilled Water for 5 min (ULT-W)
4	Calibra Try-in Paste (C)		Rinse and Dry + Ultrasonic Bath in Distilled Alcohol for 5 min (ULT-A)
5	Variolink Try-in Paste (V)	Ivoclar Vivadent, Schaan, Liechtenstein	Air–Water Spray or Rinse and Dry (RD)
6	Variolink Try-in Paste (V)		Rinse and Dry + Ultrasonic Bath in Distilled Water for 5 min (ULT-W)
7	Variolink Try-in Paste (V)		Rinse and Dry + Ultrasonic Bath in Distilled Alcohol for 5 min (ULT-A)
8	RelyX Veneer Try-in Paste (R)	3M, Neuss, Germany	Air–Water Spray or Rinse and Dry (RD)
9	RelyX Veneer Try-in Paste (R)		Rinse and Dry + Ultrasonic Bath in Distilled Water for 5 min (ULT-W)
10	RelyX Veneer Try-in Paste (R)		Rinse and Dry + Ultrasonic Bath in Distilled Alcohol for 5 min (ULT-A)

**Table 2:** Summary of residual paste removal efficacy for different try-in pastes using various cleaning methods.

Try-in Paste	Cleaning Method	Amount of Residual Paste	Surface Coverage
Control (No Contamination)	N/A	None	0%
Calibra Try-in Paste	RD	Very High	>75%
	ULT-W	Low	<25%
	ULT-A	Very High	>75%
Variolink Try-in Paste	RD	Low	<25%
	ULT-W	None	0%
	ULT-A	None	0%
Rely-X Try-in Paste	RD	None	0%
	ULT-W	None	0%
	ULT-A	None	0%

## Data Availability

The original contributions presented in the study are included in the article, further inquiries can be directed to the corresponding authors.
